# Variation in chromosome number and breeding systems: implications for diversification in *Pachycereus pringlei* (Cactaceae)

**DOI:** 10.3897/CompCytogen.v12i1.21554

**Published:** 2018-02-14

**Authors:** Carina Gutiérrez-Flores, José L. León-de la Luz, Francisco J. García-De León, J. Hugo Cota-Sánchez

**Affiliations:** 1 Department of Biology, University of Saskatchewan, 112 Science Place, Saskatoon, SK, S7N5E2, Canada; 2 HICB Herbarium, Centro de Investigaciones Biológicas del Noroeste, Instituto Politécnico Nacional 195, Playa Palo de Santa Rita Sur, C.P. 23096, La Paz, B.C.S., México; 3 Laboratorio de Genética para la Conservación, Centro de Investigaciones Biológicas del Noroeste, Instituto Politécnico Nacional 195, Playa Palo de Santa Rita Sur, C.P. 23096, La Paz, B.C.S., México

**Keywords:** Diploid, Cactaceae, cryptic speciation, cytogeography, karyotype, *Pachycereus pringlei*, polyploidy

## Abstract

Polyploidy, the possession of more than two sets of chromosomes, is a major biological process affecting plant evolution and diversification. In the Cactaceae, genome doubling has also been associated with reproductive isolation, changes in breeding systems, colonization ability, and speciation. *Pachycereus pringlei* (S. Watson, 1885) Britton & Rose, 1909, is a columnar cactus that has long drawn the attention of ecologists, geneticists, and systematists due to its wide distribution range and remarkable assortment of breeding systems in the Mexican Sonoran Desert and the Baja California Peninsula (BCP). However, several important evolutionary questions, such as the distribution of chromosome numbers and whether the diploid condition is dominant over a potential polyploid condition driving the evolution and diversity in floral morphology and breeding systems in this cactus, are still unclear. In this study, we determined chromosome numbers in 11 localities encompassing virtually the entire geographic range of distribution of *P. pringlei*. Our data revealed the first diploid (2n = 22) count in this species restricted to the hermaphroditic populations of Catalana (ICA) and Cerralvo (ICE) Islands, whereas the tetraploid (2n = 44) condition is consistently distributed throughout the BCP and mainland Sonora populations distinguished by a non-hermaphroditic breeding system. These results validate a wider distribution of polyploid relative to diploid individuals and a shift in breeding systems coupled with polyploidisation. Considering that the diploid base number and hermaphroditism are the proposed ancestral conditions in Cactaceae, we suggest that ICE and ICA populations represent the relicts of a southern diploid ancestor from which both polyploidy and unisexuality evolved in mainland BCP, facilitating the northward expansion of this species. This cytogeographic distribution in conjunction with differences in floral attributes suggests the distinction of the diploid populations as a new taxonomic entity. We suggest that chromosome doubling in conjunction with allopatric distribution, differences in neutral genetic variation, floral traits, and breeding systems has driven the reproductive isolation, evolution, and diversification of this columnar cactus.

## Introduction

Polyploidy and hybridisation are major biological events in plant evolution and speciation (Grant 1981, Wendel and Doyle 2005), often leading to complex patterns of genetic diversity, reproductive isolation, and discrepancy in breeding systems (De Wet 1971, Tate et al. 2005, Marques et al. 2014). Therefore, studies focusing on changes in chromosome numbers are instrumental to identify reproductive variability and distribution of different cytotypes at the intra- and inter-population levels and to make inferences about the origins of polyploids.

The Cactaceae, a family with approximately 1,430 species (Hunt et al. 2006), exhibits an extensive habitat radiation and reproductive versatility linked to striking specialized floral morphology (Cota-Sánchez and Croutch 2008, Almeida et al. 2013) and variation in chromosome numbers (Cota 1991, Baker et al. 2009a). As stated by Stebbins (1950, p. 369), “polyploidy ... is one of the quickest biological process producing totally different and more vigorous and well-adapted genotypes.” In the same way, polyploidy, along with variation in breeding systems, has been considered an important factor directing the evolutionary history and disparity of the Cactaceae, often resulting in the formation of new species (Baker and Pinkava 1987, 1999, Baker 2002, 2006). Remarkably, approximately 28% (154 out of 551 species) of cacti cytologically investigated have increased genome dosage, primarily in subfamily Opuntioideae (Rebman and Pinkava 2001). Genome doubling prompts the evolution of some sexual systems in the Cactaceae, i.e., gynodioecy and trioecy (Rebman and Pinkava 2001), and additional chromosome sets have been correlated with physiology and differences in morphological and geographic distribution. For example, polyploidy in cacti allows the adaptation to freezing temperatures (Cota-Sánchez 2002), taxonomic diversification (Majure et al. 2012), the colonization of higher latitudes (Cota and Philbrick 1994), wider geographical range (Barthlott and Taylor 1995, Cota-Sánchez and Bomfim-Patrício 2010), and acts as a predictor of responses to environment and evolution (Segura et al. 2007). However, for many taxa with wide ecological and geographic distribution the role and extent of polyploidy is still unknown because different ploidy levels come to light only after a cytological survey has been made across populations in an extensive geographic area.

Surveys of chromosome variation, both numerical and structural, have been successfully applied in systematic studies of the Cactaceae (Pinkava et al. 1977, Pinkava and Parfitt 1982, Mazzola et al. 1988). Chromosomal structural rearrangements in the cactus family vary from translocations in *Opuntia leptocaulis* de Candolle, 1828 (Pinkava et al. 1985) and inversions in *O. curvospina* Griffiths, 1916 (Pinkava et al. 1973) to cryptic structural changes in *Pyrrhocactus* (A. Berger, 1929) Backeberg et F.M. Knuth, 1935 (Las Peñas et al. 2008) and stable nuclear content of DNA in species of *Mammillaria* Haworth, 1812 (Christian et al. 2006). Similarly, analyses of meiotic chromosome behavior and polyploidy have been effective in addressing taxonomic problems in several genera and the hybrid status of Opuntia × spinosibacca M.S. Anthony, 1956 (Pinkava and Parfitt 1988). The natural history of cacti has also involved karyotypic studies to clarify species boundaries, the correlation of geographic range with ploidy levels and morphology, and phylogenetic relationships (Beard 1937, Grant and Grant 1979, Parfitt 1987, Cid and Palomino 1996, Das 1999, Baker and Johnson 2000, Baker and Cloud-Hughes 2014, Stock et al. 2014, Wellard 2016). Yet, there are still numerous cacti for which cytological information remains unknown.

The columnar cactus *Pachycereus pringlei* (S. Watson, 1885) Britton & Rose, 1909, has been an excellent model plant for ecological and evolutionary studies because this species has an extensive distribution range in the Mexican portion of the Sonoran Desert (Turner et al. 1995, Drezner and Lazarus 2008). Unlike most cacti, this taxon exhibits variation in genders and breeding systems (Fleming et al. 1998, Medel-Narváez 2008, Gutiérrez-Flores et al. 2016, 2017). While the vegetative morphological variability in this species is seemingly conservative to the extent that the species can be easily recognized by these attributes, the existence of polymorphism in floral traits associated with breeding systems and geographic distribution of populations is highly diverse, suggesting reproductive isolation (Gutiérrez-Flores et al. 2017). Recent studies of neutral genetic variation (Gutiérrez-Flores 2015, Gutiérrez-Flores et al. 2016) identified five genetic populations of *P. pringlei* unexpectedly associated with different breeding systems, namely two hermaphrodite populations restricted to Catalana and Cerralvo Islands in the Gulf of California, a mainly dioecious assemblage in Cabo San Lucas (CBS) at the tip of the BCP, another trioecious cohort from CBS to northern BCP (~28°N), and a mostly gynodioecious population in northern BCP and the coast of Sonora in mainland Mexico.

The biogeographic distribution pattern of *P. pringlei* has been driven by longstanding climatic fluctuations associated with differential colonization abilities of genders, geographic variation of selfing and outcrossing rates, and the effect of biotic and abiotic factors (Gutiérrez-Flores et al. 2016, 2017). As a result, the spatial segregation of genders in *P. pringlei* has long been a magnet to ecologists, geneticists, and systematists (Fleming et al. 1994, 1998, Molina-Freaner et al. 2003, Gutiérrez-Flores et al. 2016, 2017). Even so, several important evolutionary questions, such as the distribution of chromosome numbers and whether the diploid condition is dominant over a polyploid condition influencing the evolution and diversity of this cactus, remain unclear.

Chromosome counts and allozyme data have revealed that *Pachycereus pringlei* is tetraploid (2n = 44), but these reports are supported by scanty evidence from northern BCP at El Rosario (Pinkava et al. 1973) and another site in mainland Mexico in Bahía Kino (Murawski et al. 1994). Consequently, chromosome numbers in the vast area of distribution in the BCP remain unexplored. Moreover, to date there is no record indicating the existence of the family’s base chromosome number (n = 11) in this species. Since the characterization of the geographic distribution and potential variability of ploidy levels is useful to gain new insights into the natural history of this long-lived cactus in connection with the distribution of reproductive systems and genetic variation, in this paper we present a survey of chromosome numbers in new and different populations of *P. pringlei* throughout the BCP and mainland Mexico. We combine cytological data with information about breeding systems and floral and genetic diversity to discuss their relationships and role in the diversification and evolution of this species. Explicitly, the goals of the study were 1) to expand knowledge about the geographic distribution and possible variation in chromosome numbers (diploid versus polyploid cytotypes) throughout the geographic range of *P. pringlei* and 2) to examine the correspondence of ploidy levels with genetic populations, floral attributes, and breeding systems. When appropriate, a discussion dealing with taxonomic implications of variation in chromosome number with respect to morphological traits is included.

## Material and methods

### The study species

**Figure 1.**
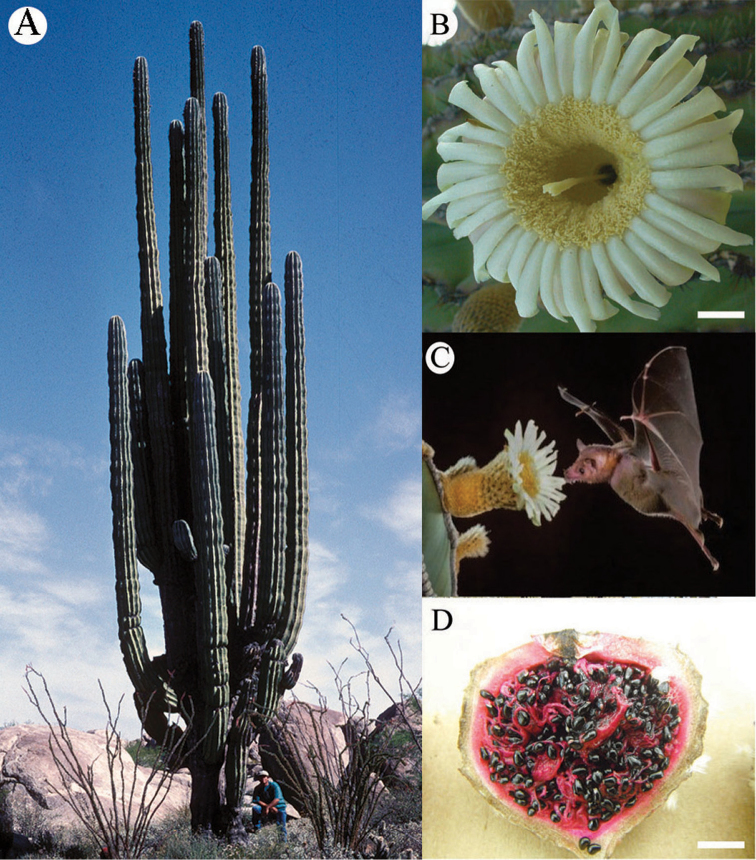
Typical vegetative and floral morphology of the emblematic cactus *Pachycereus pringlei.* **A** Mature individual with candelabra-like structure in the Cataviña region. **B** Archetypal funnel-form flower. **C** Main pollinator, the long-nosed bat *Leptonycteris yerbabuenae*. **D** Mature, fleshy fruit. Photo **A** by Jon Rebman; photo **C** by Merlin D. Tutle.

*Pachycereus pringlei*, a cactus commonly known as Cardón, is circumscribed within the subfamily Cactoideae. The species dominates rocky slopes and alluvial plains in the deserts of the BCP, most islands of the Gulf of California, and coastal areas of mainland Sonora, Mexico (Turner et al. 1995, Gutiérrez-Flores et al. 2017). Old plants reach an average height of eight to nine m, have an impressive candelabra-like shape (Fig. 1A), and bear from a few to up to 30 large branches (Bravo-Hollis and Sánchez-Mejorada 1991). The flowering season is from late March to early June with a peak from late April to mid-May. The flowers are white to cream in color (Fig. 1B) with abundant nectar and pollen, open early in the evening, and are pollinated by the long-nosed bat *Leptonycteris yerbabuenae* Martínez & Villa-R., 1940 (Phyllostomidae: Glossophaginae (Fig. 1C); however, the blossoms persist open for several hours the next morning allowing visits from diurnal pollinators, such as birds and insects, mainly bees (Fleming et al. 1996, 1998). The large, fleshy fruits with red pulp (Fig. 1D) attract frugivorous animals, facilitating seed dispersal.

### Inspection of chromosome numbers

The chromosome numbers inspected in this study were obtained from individual plants from natural populations across the wide distributional range of *P. pringlei* encompassing variation in ecology, latitude, and longitude as well as floral morphology, breeding systems, and levels of genetic diversity. Fruits with mature seeds were collected in the field from three to 10 individuals in the following localities of the BCP: Bahía de Los Ángeles (BAN), Cabo San Lucas (CBS), Catalana Island (ICA), Cerralvo Island (ICE), El Comitán (COM), López Mateos (LMA), Loreto (LOR), San Felipe (SFE), Puente Querétaro (PQU), and Santa Rosalía (SRO). Also, one fruit collected in mainland Mexico from one locality, Álamos (ALA), Sonora, was scrutinised (Table 1). We also included one previous count by Pinkava et al. (1973) from El Rosario (ROS) and an allozymatic inference of the ploidy level by Murawski et al. (1994) from Bahía Kino (BKI) (Table 1) for a grand total of 13 locations. The distance among the different sample sites varies from 20 km between López Mateos (LMA) and Puente Querétaro (PQU) to 983.7 km between Cabo San Lucas (CBS) and San Felipe (SFE).

**Table 1.** Sample sites of the columnar cactus *Pachycereus pringlei* for which chromosome numbers were investigated, including counts by Pinkava et al. 1973 (†) and Murawski et al. 1994 (*). All diploid counts represent new reports for this species. Breeding systems, genetic diversity and genetic populations according to Gutiérrez-Flores et al. (2016). ND = not determined.

Locality	Code	Latitude	Longitude	Chromosome number	Ploidy level	Breeding system	Genetic diversity	Genetic population
Cabo San Lucas	CBS	22.9438, -109.9905		2n = 44	Tetraploid	Mainly dioecious	0.38	CBS
Catalana Island	ICA	25.6768, -110.8087		2n = 22	Diploid	Hermaphroditic	0.40	ICA
Cerralvo Island	ICE	24.1868, -109.8775		2n = 22	Diploid	Hermaphroditic	0.26	ICE
El Comitán	COM	24.1332, -110.4317		2n = 44	Tetraploid	Mainly trioecious	0.45	South
López Mateos	LMA	25.2726, -111.8942		2n = 44	Tetraploid		0.45	
Loreto	LOR	25.8918, -111.4698		2n = 44	Tetraploid		ND	
Puente Querétaro	PQU	25.3508, -111.6094		2n = 44	Tetraploid		0.45	
Santa Rosalía	SRO	27.2408, -112.3615		2n = 44	Tetraploid	Mainly gynodioecious	ND	North
Bahía de Los Ángeles	BAN	28.9164, -113.5541		2n = 44	Tetraploid		0.35	
El Rosario	ROS	30.0861, -115.6795		n = 22	Tetraploid†		ND	
San Felipe	SFE	30.3716, -114.8537		2n = 44	Tetraploid		0.35	
Álamos	ALA	26.8978, -109.4578		2n = 44	Tetraploid		0.35	
Bahía Kino	BKI	28.5000, -111.8063		ND	Tetraploid*		ND	

Mitotic chromosome numbers were determined using meristematic cells from fresh root tips following a modified protocol by Cota et al. (1996). Approximately 30 seeds per fruit were first rinsed with 10 % commercial bleach (NaClO) and then germinated in Petri dishes with moistened filter paper under controlled greenhouse conditions. The one-week-old root tips of ca. 1 cm in length were trimmed and immersed in Colchicine 0.2 % to arrest chromosomes at metaphase and kept at 4 °C for 2–4 h, then rinsed twice with distilled water and fixed in Carnoy’s fluid (3:1 ethanol 95 % and glacial acetic acid v/v) overnight. Next, the roots were rinsed with distilled water, hydrolysed in 1N HCl at 60 °C, rinsed twice with distilled water, and stained with aceto-orcein at room temperature for 1 h. Semi-permanent slides were prepared by squashing root tips in Hoyer’s medium and then examined in a Zeiss Axio Imager Z1 microscope (Carl Zeiss, Toronto, ON, Canada) at 40×, 60×, and 100× (immersion oil). Photographs were taken using an AxioCam MRm charge-coupled digital CCD camera and AxioVision 4.8 imaging software. Chromosome size was estimated using the measuring tool available in the AxioVision 4.8 imaging software to add the corresponding scale bar to the pictures. For consistency, meiotic chromosome counts in CBS were also performed in anthers from mature flower buds of one hermaphrodite and two male plants.

For meiotic figures and counts, anthers from floral buds at different developmental stages were fixed in Carnoy’s solution, then stained with aceto-orcein at room temperature for 1 h, squashed, mounted in Hoyer’s medium and microscopically examined as indicated above. A minimum of three cytological figures from different individuals were scrutinized in each population for confirmation of chromosome number. Finally, the geographic distribution of diploid and polyploid cytotypes from the localities examined was plotted on a base map obtained from the Comisión Nacional para el Conocimiento y Uso de la Biodiversidad (CONABIO, http://www.conabio.gob.mx) using the ArcGIS 10.4 software (ESRI).

### Idiogram construction

Idiograms were reconstructed based on microscopic observation of mitotic figures. Chromosome homology for diploid and tetraploid cytotypes follows Cota and Wallace (1995), which is based on similarities in morphology, length, and centromere position, the primary physical features used because no satellites or secondary constrictions were detected.

### Phenotypic variation in floral characters

To compare floral variability among diploid and polyploid populations of *P. pringlei*, 17 floral traits were selected and a one-way ANOVA of morphological attributes from flowers gathered for the five genetic populations *fide* Gutiérrez-Flores et al. (2017) was performed. Only data from bisexual flowers were used to avoid potential misinterpretations due to gender variation. Measurements of floral phenotypic traits and ANOVA tests are summarized in Table 2.

**Table 2.** Measurements of phenotypic floral traits, including sample size (mean ± SE) and statistical comparisons of bisexual flowers between diploid (ICE, ICA) and polyploid (CBS, SOUTH, NORTH) populations of *Pachycereus pringlei* in the Baja California Peninsula, Mexico. Lower case superscript letters indicate floral characters having statistically significant differences.

Floral trait	Diploid		Polyploid		
	ICE	ICA	CBS	SOUTH	NORTH
	n = 50	n = 35	n = 7	n = 20	n = 38
Corolla width (mm)	33.3 ± 0.7	36.2 ± 0.9	29.4 ± 1.2	33.1 ± 1.0	36.0 ± 0.7
Floral length (mm)	90.7 ± 1.2	86.8 ± 0.4	76.4 ± 2.8	85.1 ± 2.1	96.2 ± 1.5
Nectary length (mm)	12.9 ± 0.3	13.0 ± 0.3	9.2 ± 0.4	11.9 ± 0.4	13.0 ± 0.3
Nectary width (mm)	11.1 ± 0.2b	10.7 ± 0.3b	8.7 ± 0.5a	9.3 ± 0.2a	9.9 ± 0.3a
No. of pollen grains (x10^6^)	2.8 ± 0.3c	2.3 ± 0.2cb	1.0 ± 0.2a	1.3 ± 0.2a	1.6 ± 0.2ab
No. of stamens (in 0.5 cm^2^)	48.6 ± 1.0ab	43.0 ± 1.8a	49.0 ± 3.14ab	53.4 ± 1.9b	51.6 ± 2.0b
No. of tepals	49.9 ± 0.6	46.7 ± 1.2	44.3 ± 1.6	47.9 ± 1.4	49.9 ± 0.8
P:O ratio (x10^3^)	2337 ± 255b	2638 ± 307b	1178 ± 289a	911 ± 117a	2166 ± 203a
Stamen length (mm)	10.0 ± 0.2	10.2 ± 0.2	11.9 ± 0.7	10.0 ± 0.2	10.9 ± 0.2
Tepal length (mm)	23.0 ± 0.4	23.5 ± 0.4	19.0 ± 0.6	20.4 ± 0.6	23.0 ± 0.5
Tepal width (mm)	7.7 ± 0.3	8.1 ± 0.3	5.9 ± 0.3	8.0 ± 0.3	9.1 ± 0.2
Ovary length (mm)	14.0 ± 0.4	9.4 ± 0.4	8.3 ± 0.7	13.4 ± 0.7	15.5 ± 0.5
Ovary width (mm)	8.9 ± 0.3	7.9 ± 0.3	8.1 ± 0.5	8.7 ± 0.4	9.6 ± 0.3
Pistil length (mm)	44.6 ± 0.7	47.1 ± 0.9	43.8 ± 1.2	47.9 ± 1.4	51.0 ± 0.9
Stigma length (mm)	9.8 ± 0.3	8.8 ± 0.3	7.7 ± 0.5	8.9 ± 0.3	10.7 ± 0.4
No. of ovules (mm)	1550 ± 66b	907 ± 40a	849 ± 115a	1505 ± 118b	1614 ± 112b
Stamen-stigma distance (mm)	0.5 ± 0.5a	2.0 ± 0.6ab	1.5 ± 1.1ab	4.2 ± 1.0bc	5.4 ± 0.6c

## Results

### Chromosome number and morphology

**Figure 2.**
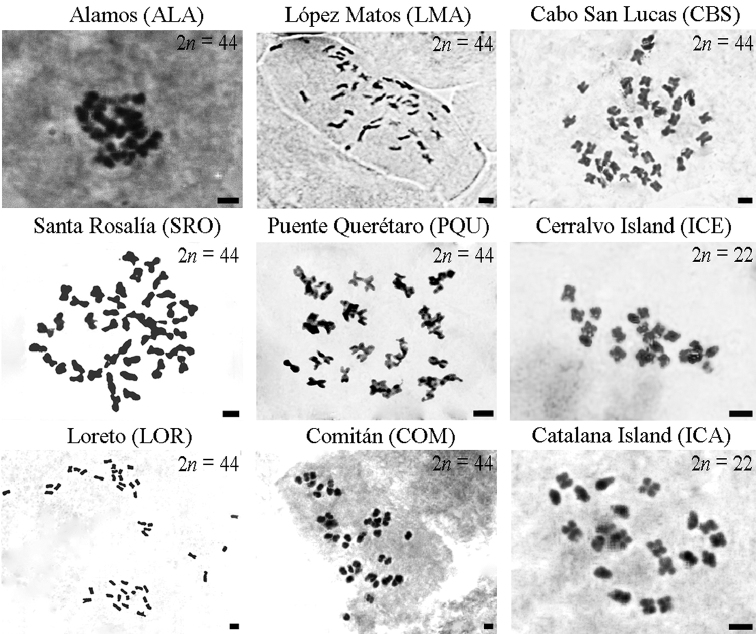
Representative metaphase chromosomes of *Pachycereus pringlei*. Cerralvo Island (ICE) and Catalana Island (ICA) are the sole localities for diploid cytotypes. Remaining pictures in plate are typical chromosomes in different tetraploid populations. Scale Bar: 2 µm.

**Figure 3.**
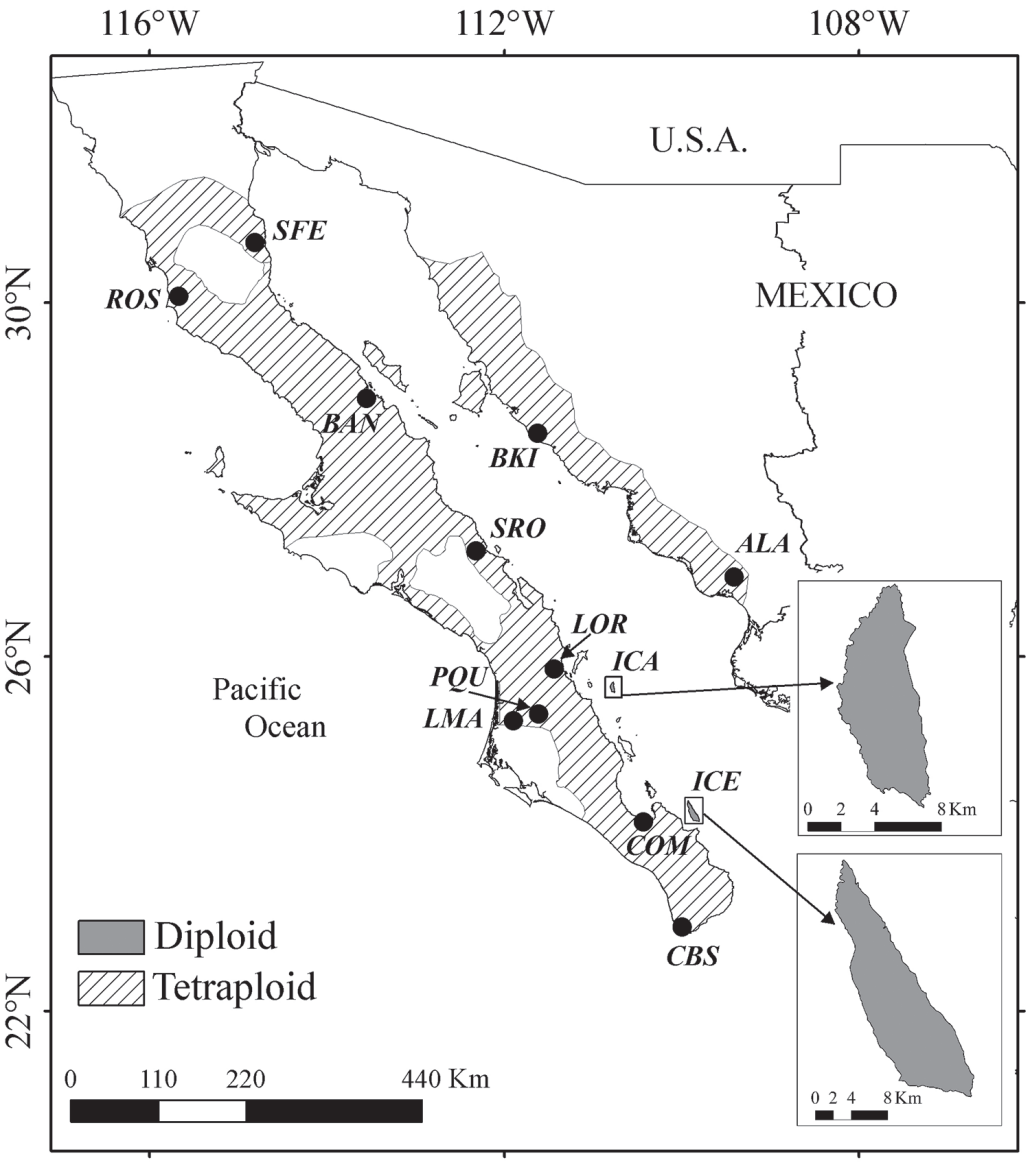
Distribution of diploid and tetraploid individuals in populations (black dots) of *Pachycereus pringlei*. Dark gray areas in ICA and ICE indicate distribution of diploid (2n = 22) cytotypes. Diagonal shaded area indicates the predicted coverage of tetraploid (2n = 44) cytotypes. See Table 1 for full names of the abbreviated localities indicated in the map.

Chromosome counts performed in the populations of *P. pringlei* investigated revealed variation in ploidy level. Foremost, our survey unveiled the first diploid (2n = 2x = 22) count for this species and expanded the current cytological knowledge with additional tetraploid (2n = 4x = 44) cytotypes in different localities of the BCP. The diploid counts were consistently determined in all mitotic cells in plants from Cerralvo (ICE) and Catalana (ICA) Islands (Figs 2, 3; Table 1), which are composed of hermaphrodite populations. Double chromosome dosage was found in all the remaining populations investigated from both the BCP and mainland Mexico (Figs 2, 3; Table 1). All counts performed in pollen grains from flower buds were diploid (n = x = 22), and no abnormal figures or disruptive cell divisions were noted. These observations supported tetraploidy in two male individuals and one hermaphrodite plant from CBS.

**Figure 4.**
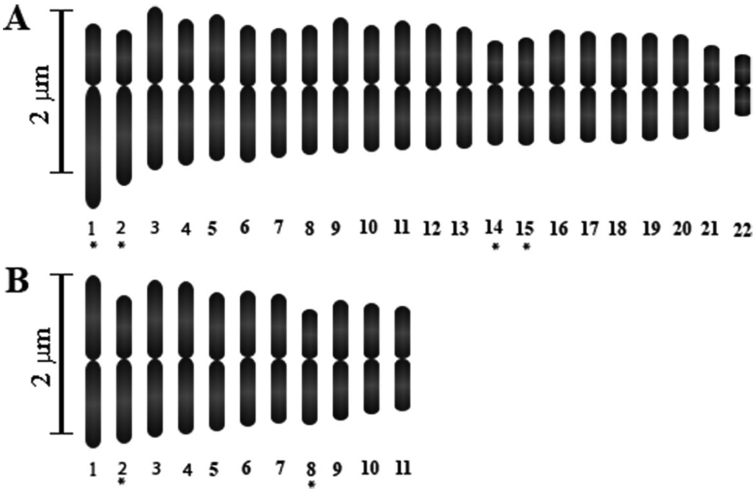
Idiograms of tetraploid (**A**) and diploid (**B**) cytotypes of the columnar cactus *Pachycereus pringlei*. Asterisk (*) denotes submetacentric chromosomes.

The overall morphology of mitotic chromosomes for diploid and tetraploid cytotypes of this columnar cactus is, in general, symmetric and uniform in shape, i.e., chromosomes are mostly metacentric (M) with a few submetacentric (SM) and relatively small in size (measuring in average 2µm in length) (Figs 2, 4). No visible physical structural differences or secondary constrictions (satellites) were detected in any of the diploid and polyploid populations investigated. The karyotype in diploid and tetraploid populations is also symmetric (Fig. 4) and the variation mainly involves numerical changes with insignificant differences in chromosome shape and proportions.

### Phenotypic variation in floral characters

Morphological comparisons of bisexual flowers among populations revealed a few significant statistical differences that can be associated with variation in ploidy level. For instance, diploid individuals from ICE and ICA have wider nectaries (11.1 ± 0.2 and 10.7 ± 0.3 mm, respectively) and larger P:O ratios (2,337 ± 255 and 2,638 ± 307, respectively). The diploid cytotypes also tend to have larger amount of pollen grains (2.8 x106 and 2.3 x106, respectively), fewer number of stamens (48.6 ± 1.0, and 43.0 ± 1.8, respectively), fewer number of ovules (1,550 ± 66 and 907 ± 40, respectively), and closer proximity between stamen and stigmas (0.5 ± 0.5 and 2.0 ± 0.6 mm, respectively). See also Table 2.

## Discussion

### Geographic and range expansion of chromosome numbers in *P. pringlei*

Chromosome number variation, especially polyploidy, is one of the major biological processes that has affected angiosperm evolution (Stebbins 1971), leading to different or new evolutionary lines promoting new genome combinations in organisms (Wendel and Doyle 2005), including the Cactaceae (Cota and Philbrick 1994). The significance of polyploidy in the cactus family is more evident in the subfamily Opuntioideae, in which polyploid taxa, primarily in the genus *Opuntia* Miller, 1754 (Pinkava and McLeod 1971, Pinkava et al. 1973, 1998, Baker and Pinkava 1987, Baker et al. 2009b, Majure et al. 2012), are common. In subfamily Cactoideae, polyploidy is more sporadic despite the large number of species circumscribed in this group, probably due to the relatively recent origin and the unexplored cytological aspects of this lineage. Different levels of polyploidy have been reported in various clades of the Cactoideae including terrestrial, e.g., *Blossfeldia liliputana* Wendermann, 1937 (Ross 1981), *Echinocereus* Engelmann, 1848 (Cota 1991, Cota and Wallace 1995), *Mammillaria* spp. (Ross 1981), *Weberbauerocereus weberbaueri* (K. Schumann ex Vaupel, 1913) Backeberg, 1958 (Sahley 1996), and epiphytic taxa, e.g., *Hylocereus* (A. Berger, 1905) Britton & Rose, 1909 and *Selenicereus* (A. Berger, 1905) Britton & Rose, 1909 (Lichtenzveig et al. 2000), and *Rhipsalis baccifera* (Solander, 1771) Stearn, 1939 (Cota-Sánchez and Bomfim-Patrício 2010). Additionally, autotetraploidy (four copies of a single genome due to doubling of an ancestral chromosome complement) has been documented in the columnar cactus *W. weberbaueri* (Sahley 1996) and in *P. pringlei* (Murawski et al. 1994).

This study has unveiled the first report of diploid (2n = 2x = 22) cytotypes in *P. pringlei* and expands the distributional range of the tetraploid (2n = 4x = 44) condition known for this species. Geographically, our survey also revealed that the base diploid number is maintained exclusively in the two deep-water islands (ICA and ICE) of the Gulf of California characterised by the prevalence of hermaphrodite individuals. In turn, double chromosome dosage (tetraploidy) is consistent throughout the three BCP populations (CBS, North and South) (Fig. 3) and is associated with the presence of unisexual plants and a dioecious, gynodioecious or trioecious breeding system (Table 1). We predict that the tetraploid condition extends to other continental islands, such as Espíritu Santo, San José, Monserrat, San Lorenzo, and Tiburón, which are areas with unisexual plants (Gutiérrez-Flores 2015). Conceivably, an increment in chromosome number has enabled *P. pringlei* to colonize wide areas of the BCP. The same premise has been proposed in other cacti, e.g., *Echinocereus*, in which polyploid taxa occupy wider territories and ecological sorting, from medium to high latitudes and elevations relative to the overall distribution of the genus and diploid relatives (Cota and Philbrick 1994, Cota-Sánchez 2008). Also, comparable biogeographic patterns exist for other angiosperm taxa with different cytotypes, e.g., *Chamerion* (Rafinesque, 1818) Rafinesque ex Holub, 1972 (Husband and Schemske 1998), *Chrysolaena* H. Robinson, 1988 (Do Pico and Dematteis 2014), and members of the Asparagaceae (Azizi et al. 2016).

Among polyploids, tetraploidy is the most successful condition (De Wet 1980). In fact, tetraploidy is the most frequent form in the Cactaceae (Pinkava et al. 1985), and the radiation success of *Echinocereus* from central Mexico to the southwest of the US has been attributed to the prevalence of tetraploids throughout the distribution range (Cota 1991, Cota and Philbrick 1994) and the *Humifusa* clade of *Opuntia* s.s. (Majure et al. 2012). Similarly, the relatively fast radiation of the South American epiphytic cactus *Rhipsalis baccifera* into areas of the New and Old Worlds has been accompanied by successive cycles of polyploidy (di-, tetra- and hexaploid), in conjunction with life history traits, such as facultative selfing, asexual reproduction, and vivipary (Cota-Sánchez and Bomfím Patrício 2010). Accordingly, the incidence of polyploidy can be associated with increasing the colonizing ability and diversification of species into new environments due to relaxed selection in the additional genome copies (Stebbins 1971, Adams and Wendel 2005, Cota-Sánchez 2008). Thus, it is not surprising that the dominance and success of polyploid (tetraploid) cytotypes in *P. pringlei* is reflected in their extensive distribution and ability to colonize a widespread range of habitats and ecological conditions in relation to the diploid state, which is restricted to ICA and ICE. Recent studies, e.g., Levy and Feldman (2004), Adams and Wendel (2005), have shown that the paramount ability of polyploids to adapt to a gamut of novel factors is based on a wide spectrum of molecular and physiological adjustments conferred by the amalgamation of two or more genomes.

During past geological events, the southern BCP, including ICE and ICA Islands, exhibited suitable niche conditions that served as refugia for populations of *P. pringlei* during the Last Glacial Maximum (LGM), as evidenced by Ecological Niche Modelling (Gutiérrez-Flores et al. 2016). Consequently, we suggest that ICE and ICA could be the relicts of a southern BCP diploid ancestor of *P. pringlei* from which both the polyploid and unisexual conditions gradually evolved in concert in mainland BCP. This, in turn, facilitated the northward range expansion during the Holocene, resulting in the modern dispersal of this neopolyploid complex with concomitant colonisation of suitable habitats available after the glacial retreat, leading to the present-day distribution of this cactus throughout the BCP and mainland Sonora. The same rationale has been used to explain the colonisation of large geographic areas of the polyploid *Opuntia humifusa* (Rafinesque, 1820) Rafinesque, 1830 s.l. and *O. macrorhiza* Engelmann, 1850 s.l. based on LGM events (Majure et al. 2012). Similarly, rapid high polyploidisation caused by recurrent population fragmentation and expansion during the Pleistocene has taken place in other angiosperms, including *Cerastium* Linnaeus, 1753, *Draba* Linnaeus, 1753, *Parnassia* Linnaeus, 1753, *Saxifraga* Linnaeus, 1753, and *Vaccinium* Linnaeus, 1753 (Abbott and Brochmann 2003, Brochmann et al. 2004), and other plants. Nonetheless, it is unclear whether the extent of polyploid populations of *P. pringlei* in the BCP is a consequence of higher diversification rates following the ubiquitous genome duplication.

### Chromosome morphology

In terms of morphology, the chromosomes of *P. pringlei* follow the overall homogeneous pattern reported for the sister taxon *P. weberi* (J.M. Coulter, 1896) Backeberg, 1960 (Gama-López 1994) and, in general, for the cactus family (Cota and Wallace 1995, Las Peñas et al. 2008 and references therein). Because of the similarity and minute size of chromosomes among cytotypes, no major structural differences were detected. That is, the chromosomes are consistently small and mostly metacentric with no evident secondary satellites. Therefore, except for the numerical difference, the homogeneous chromosome morphology in terms of arm length and shape makes the characterization between diploid and tetraploid cytotypes difficult. In addition to uniformity, chromosome size between diploid and tetraploid individuals is also insignificant, and structural changes, if any, remain cryptic. Similarly, in *Opuntia*, morphological differentiation is equivocal and has been a commonly reported event among diploid and polyploid cytotypes (Majure et al. 2012). Thus, structural chromosomal arrangements, either cryptic or physical, should not be ruled out in this columnar cactus because speciation without detectable chromosomal changes or divergence has been documented in other plants, e.g., *Platanus* Linnaeus, 1753 (Swanson et al. 1981) and *Stephanomeria* Nuttall, 1841 and *Clarkia* Pursh, 1814 (Crawford 1985).

Stebbins’ (1971) hypothesis on the frequency of chromosome types in plants is useful to explain the existence of symmetric idiograms in diploid and tetraploid cytotypes of *P. pringlei*. In plants, metacentric chromosomes are fairly common, e.g., Araceae (Turco et al. 2014), Arecaceae (Oliveira et al. 2016) and Asparagaceae (Chen et al. 2017), and originate by the fusion of two telocentric chromosomes with relatively little effect in gene sequence (Stebbins 1971). In the Cactaceae, symmetric karyotypes are also ordinary, e.g., *Echinocereus* (Cota and Wallace 1995), *Nyctocereus* (A. Berger, 1905) Britton & Rose, 1909 (Palomino et al. 1988), *Setiechinopsis* Backeberg, 1950 (Las Peñas et al. 2011), Opuntia Ser. Armata (Las Peñas et al. 2017), and other species exhibiting low degree of variability in karyotype morphology. Hence, the morphological homogeneity of chromosomes in *P. pringlei* is not surprising.

### Chromosome number and diversification of breeding systems

Foremost, it is worth noting that the correspondence of the base chromosome number (x = 11) with a hermaphroditic mating system in most cacti, including members of the basal subfamily (Pereskioideae) and species closely related to *P. pringlei*, such as *P. weberi* (Gama-López, 1994) and *P. pecten-aboriginum* (Engelmann ex S. Watson, 1886) Britton & Rose, 1909 (Pinkava et al. 1977), indicates that both the evolution of unisexuality and polyploidy are derived conditions in the Cactaceae, an idea put forward by Gutiérrez-Flores et al. (2017). In fact, there is an apparent transition between breeding system and ploidy level. The change from hermaphroditism to trioecy is coupled with an increase in chromosome number in *P. pringlei* and is consistent with polyploidisation events reported for other cacti with non-hermaphroditic sexual systems (Valiente-Banuet et al. 1997, Fleming et al. 1998, Strittmatter 2002, Díaz and Cocucci 2003, Strittmatter et al. 2006, 2008). Our results imply that in *P. pringlei* the hermaphrodite system is diploid and restricted to ICA and ICE, whereas the tetraploid condition is essentially associated with unisexual, dioecious, gynodioecious and trioecious breeding systems (Table 1). Also, the high level of genetic divergence reported for the ICE and ICA populations (Gutiérrez-Flores et al. 2016) support the general idea that difference in ploidy level is an important factor for reproductive isolation, as proposed by Baker and Pinkava (1987, 1999), Cota and Philbrick (1994), and Baker (2002, 2006). However, intra- and inter-population experimental crosses between different cytotypes are needed to determine the degree of compatibility and reproductive potential.

### Taxonomic implications in relation to morphology and ploidy level

Diploid and tetraploid plant populations may or may not be ecologically differentiated (Cota 1991). According to De Wet (1980), in the absence of chromosome information, close morphological resemblance may imply genetic continuity, a characteristic of conspecific populations. However, when a difference in ploidy level is known to exist, the issue is that barriers to gene exchange characteristic of distinct species are generally found between diploid and polyploid populations, which poses a taxonomic problem, i.e., whether or not to recognize these entities as two different species on the basis of reproductive isolation due to differences in chromosome number.

Unveiling polyploid individuals from diploid ancestors leads to the discovery of new cytotypes and potential taxonomic issues because different populations are frequently associated with an assortment of floral and/or vegetative phenotypes (Brickford et al. 2007). In this regard, the phenotypic variation reported in vegetative (Medel-Narváez et al. 2006) and reproductive (Gutiérrez-Flores et al. 2017) traits in populations of *P. pringlei* has been interpreted as a physiological response to a gradual change in environmental conditions and sex-specific selection acting at different magnitudes on sexual characters of floral morphs and populations (Gutiérrez-Flores et al. 2017). However, considering floral attributes (Table 1) and data reported by Gutiérrez-Flores et al. (2017), it is clear that the flowers from the diploid populations in ICA and ICE have, in general, wider nectaries, shorter stamen-stigma distance, larger amounts of pollen grains, larger P:O ratios, and fewer number of stamens and ovules compared to flowers of the polyploid counterparts. These morphological differences in conjunction with discrepancy in chromosome number, and geographic isolation are key elements suggesting the description of a new subspecies. Although some may argue that these few, somewhat obscure, floral traits may not warrant the recognition of a new taxonomic entity even with the evidence of a difference in ploidy, we argue that these floral features play a role in pollinator selection and breeding systems, reinforcing reproductive isolation between polyploids in mainland BCP and Sonora and their diploid progenitors in populations from the ICA and ICE islets, further suggesting a distinct taxonomic unit. Thus, rather than one species with different cytotypes, which can hinder insights into evolution, speciation, and conservation, we propose two genetically divergent subspecies with distinct ploidy levels, geographic ranges, breeding systems, and floral morphological differences. In concert, all these factors may also be largely responsible for the genetic divergence and putative reproductive isolation between diploid and tetraploid *P. pringlei* populations. This idea is also substantiated by several cytological studies providing compelling evidence to effectively distinguish diploid from polyploid species of cacti by correlating morphology with geography. For instance, the existing geographic variation of diploid and tetraploid phenotypically similar cytotypes in *Echinocereus* spp. (Cota and Philbrick 1994), the morphological disparities, sometimes cryptic, among diploid and polyploid cytotypes of the *Humifusa* clade of *Opuntia* s.s. (Majure et al. 2012), the dimorphic hexaploid (2n = 66) *Echinocereus yavapaiensis* M.A. Baker, 2006 (Baker 2006), as well as in other higher plants (Bickford et al. 2007, Rani et al. 2015, Molgo et al. 2017) and animals (Vacher et al. 2017) have been used as evidence to delimit new taxa.

Evidently, genomic changes potentially produce new gene complexes, facilitating rapid evolution of individuals and their new attributes (Soltis and Soltis 1999). However, although some functional traits are important in explaining species success, genome flexibility, and versatility in reproductive systems, morphological evaluations encompassing a large, wide-ranging number of individuals are needed to deal with a formal taxonomic description and the implications arising from differences in ploidy and patterns of geographic variation and inconsistency in morphological features throughout the populations of *P. pringlei*. At present, we can only say that variations in morphology, genetic diversity, and ploidy level suggest reproductive isolation and support the recognition of a new taxonomic entity.

## Concluding remarks

Merging chromosome number information, genetic data, breeding systems, and floral morphology has provided new insights to better understand the evolutionary history and reproductive success of this iconic cactus in northwestern Mexico. *P. pringlei* has diploid and tetraploid populations with distinctive distribution. Although tetraploids have not been named as distinct species due to the tradition of including multiple cytotypes derived from diploid relatives as a single species and the practicality of adhering to the general morphological species concept (Soltis et al. 2007), our results allude to the possibility of describing a new subspecies in *P. pringlei*. The diploid condition is endemic to Catalana and Cerralvo Islands, whereas polyploid populations characterise the populations from mainland BCP and Sonora. This cytogeographic distribution suggests the distinction of the diploid populations as a new taxonomic entity, which is likely the ancestral condition of the broadly distributed tetraploid throughout the BCP. Succinctly, we suggest that chromosome doubling in conjunction with allopatric distribution, differences in neutral genetic variation, floral traits, and breeding systems has driven the reproductive isolation, evolution, and diversification of this columnar cactus. These functional attributes render this species an ideal candidate to conduct ecological genetic investigations to further explore the selective forces acting on plants and their life history traits.
